# Impact on physical and mental health among medical personnel in Wuhan during COVID-19 outbreak: a cluster analysis

**DOI:** 10.7150/ijms.51315

**Published:** 2021-01-11

**Authors:** Jing Gao, Jing Li, Xia Han, Yuan Yuan, Chuan-Xing Li, Dong-Quan Zhang

**Affiliations:** 1 Department of Respiratory Medicine, Gansu Provincial Hospital, Lanzhou, China.; 2 Respiratory Medicine Unit, Department of Medicine & Centre for Molecular Medicine, Karolinska Institutet, Stockholm, Sweden.; 3 Heart and Lung Centre, Department of Pulmonary Medicine, University of Helsinki and Helsinki University Hospital, Finland.; 4 Department of Cardiology Medicine, Gansu Provincial Hospital, Lanzhou, China.; 5 Department of Internal Medicine, Gansu Provincial Hospital, Lanzhou, China.; 6 Department of Intensive Care, Gansu Province Hospital, Lanzhou, China.

**Keywords:** COVID-19, medical personnel, physical healthy, mental health, cluster

## Abstract

**Background:** Increased stress among medical personnel had been reported in previous virus outbreaks. The novel coronavirus disease (COVID-19) emerged in December 2019, caused by severe acute respiratory syndrome coronavirus 2 (SARS-CoV-2). No qualitative assessment has yet described the physical and mental health conditions of frontline medical personnel in the COVID-19 outbreaks.

**Methods:** Here, 251 frontline medical personnel involved in COVID-19 missions completed electronic questionnaires, consisting of 31 categorical variables related to their physical and mental health status, medical history and environmental conditions. We constructed a correlation amongst these variables through pairwise Kendall rank correlation coefficient test. Then, clusters of highly correlated variables were identified using the leading eigenvector. Finally, we used the network and clusters to clarify the correlations amongst variables.

**Results:** This qualitative study identified the six clusters. Cluster 1 was characterized by skin allergy. Cluster 2 was predominantly associated with anxiety. Cluster 3 consisted mostly of respiratory symptoms. The participants in cluster 4 had medical history. Cluster 5 and cluster 6 were characterized by disinfection and demography, respectively. Finally, we revealed three major findings. First, more than 80% of medical personnel worry about COVID-19-related infection and experience newly appearing anxiety (56.2%), airway or heart symptoms (34.3%) and skin allergies (20.3%). Second, COVID-19-related worry significantly associates with all variables in the anxiety and respiratory symptom clusters. Third, new-onset skin allergies did not associate with either disinfection or anxiety, but did associate with a previous history of allergies.

**Conclusions:** COVID-19-related worry leads to physical and mental health problems amongst medical personnel. Effective responses and interventions could relieve a series of new-onset physical and mental health problems.

## Introduction

The novel coronavirus disease (COVID-19) emerged in December 2019 in Wuhan, Hubei Province in China 1, caused by severe acute respiratory syndrome coronavirus 2 (SARS-CoV-2). Since then, more than 40 000 Chinese medical personnel outside of Hubei Province have supported the Hubei medical system 2. The previous studies showed that the mental health of medical and nursing staff met greatly challenged during the immediate wake of the viral epidemic 3,4. At recent, it reports that medical personnel experience mental health disturbed during COVID-19 outbreak 5. However, no qualitative data currently exists on the physical and mental health among medical personnel during COVID-19. Hence, to explore the impact on physical and mental health status and classify the parameters among medical personnel during COVID-19 outbreak, we conducted a qualitative study.

## Methods

An electronic questionnaire was designed based on the literature and suggestions from experts. The medical aid team from Gansu Province, China, consisting of doctors, nurses and centre for disease control (CDC) personnel participated in this study. This team worked in Wuhan for the anti-COVID-19 aid mission beginning at the end of January 2020. They answered the questionnaire between 3 and 7 March 2020. In total, 31 categorical variables from 251 participants were used to evaluate the physical and mental health status, medical history and environmental conditions amongst participants. First, we performed tests of pairwise Kendall rank correlation coefficients, further applying the Bonferroni adjustment of multiple testing corrections. Then, significant (false discovery rate (FDR) <0.1) correlated variable pairs were used to construct an edge-weighted (weight = -log10 (p value)) network. We identified clusters of highly correlated variables using the leading eigenvector in R package *igraph*. The Ethics Committee of the Gansu Province People's Hospital approved the study protocol (No. 2020-018), and all participants provided their written informed consent.

## Results

**Figure 1:**
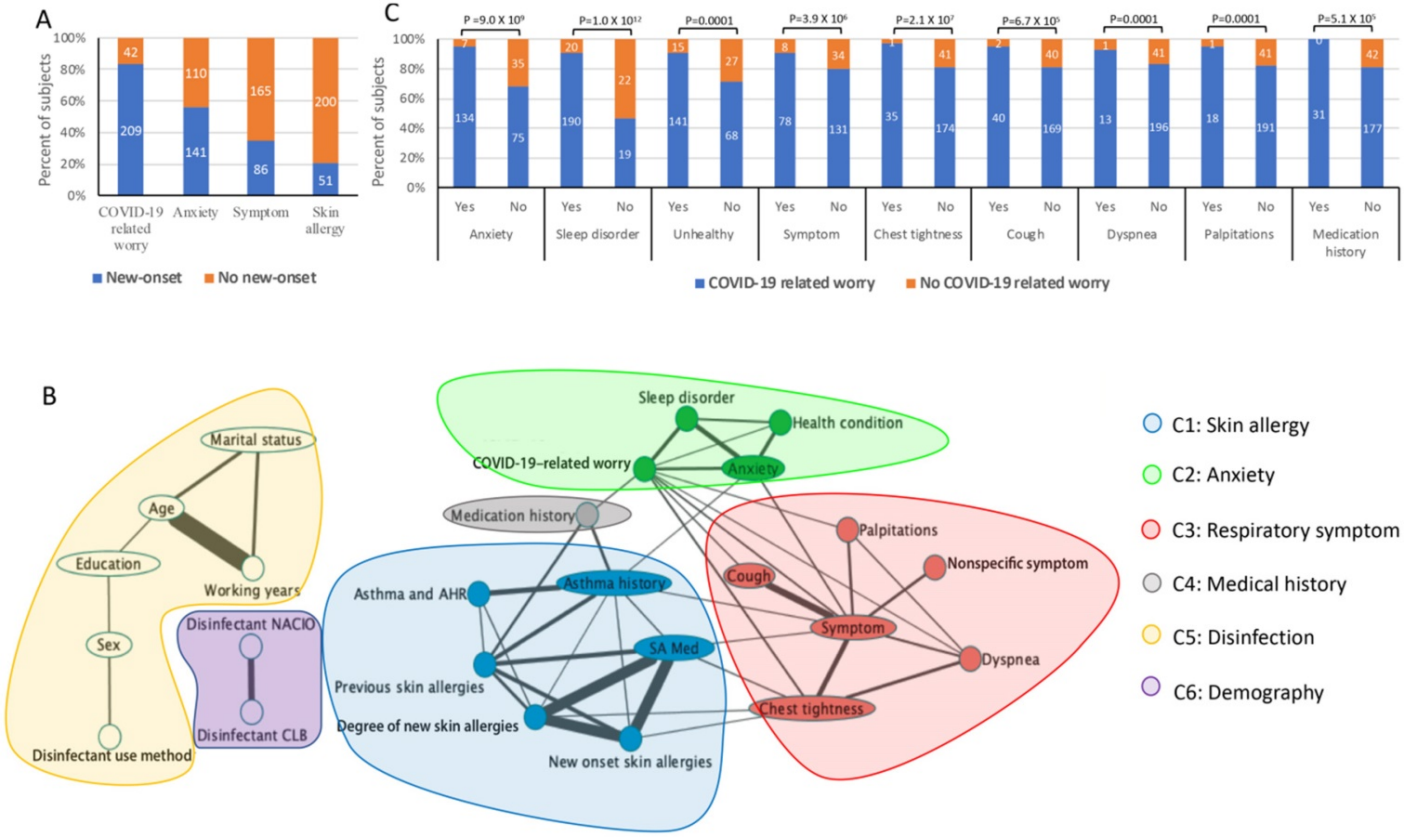
** Assessment of the physical and mental health condition of medical personnel during COVID-19 outbreak.** Note: (A). Percentage (y-axis) of new-onset physical and mental health issues (x-axis). (B). Association between COVID-19-related worry and linked variables along the x-axis. The y-axis shows the percentage of subjects with or without COVID-19-related worry by colour. (C). Six clusters (colours in the legends) of physical and mental health conditions amongst medical personnel during COVID-19 outbreak. Nodes represent categorical variables in the questionnaire. Significant pairs of variables identified from the Kendall rank correlation test (FDR≤0.1) are connected to the edge of the width representing the significance (-log10(p value)). SA Med, skin allergy medication; NACIO, sodium hypochlorite disinfectant; CLB, chlorine-based disinfectant.

Amongst 251 medical staff ranging in age from 22 to 54 years, 202 (80.5%) were female, 219 (87.3%) had more than 6 years of work experience and 23 (9.2%) had a history of asthma or airway hyper reactivity (AHR). A total of 83.7% subjects worried about contracting COVID-19 (COVID-19-related worry, Figure 1A), 83.3% of whom expressed mild worry. Furthermore, 56.2% of participants felt anxious (Figure 1A), 97.9% of whom experienced mild anxiety. In addition, 34.3% of participants experienced new airway or heart symptoms, the typical signs of which consisted of a cough and chest tightness, and 20.3% subjects experienced new-onset skin allergies (Figure 1A), primarily manifested as a rash and itching. All participants disinfected their living areas at least once daily, reporting on the frequency, method of disinfection (spray or wipe) and the type of disinfectant used.

Overall, 49 of 465 pairwise correlations were significant (FDR≤0.1) and were further merged into an association network in which we identified six clusters of significantly correlated variables (Figure 1B). Cluster 1 (C1) was characterized by skin allergy. Cluster 2 (C2) was predominantly associated with anxiety. Cluster 3 (C3) consisted mostly of respiratory symptoms. The participants in cluster 4 (C4) had medical history. Cluster 5 (C5) and cluster 6 (C5) were characterized by disinfection and demography, respectively. New-onset skin allergy did not associate with either disinfection or anxiety, which was supported through the isolation between clusters of skin allergies (C1) and disinfection (C5) or anxiety (C2). A skin allergy was 5.5 times more likely to appear in participants with a history of a skin allergy than amongst those without it. Similarly, a skin allergy was 2.7 times more likely to appear in the group reporting a history of asthma and 2.7 times more likely in subjects with new-onset chest tightness syndrome. C3 included five loosely associated new-onset syndrome variables consisting of a cough, chest tightness, palpitations and other nonspecific self-reported symptoms. Notably, COVID-19-related worry significantly associated with all of the variables in the anxiety cluster and the respiratory syndrome cluster (Figures 1B and 1C), but not with the skin allergy cluster. COVID-19-related worry was 1.4 times more likely to appear in the anxiety group and 1.2 times more likely in the cough group (Figure 1C).

## Discussion

This study identifies the mental and physical conditions of medical personnel in the anti-COVID-19 work environment in Wuhan. These results identify three primary findings. First, more than 80% of medical personnel worried about contracting COVID-19-related infection as well as newly appearing anxiety (56.2%), airway or heart symptoms and skin allergies. Second, COVID-19-related worry significantly associated with all of the variables in the anxiety and respiratory symptoms clusters. Third, new-onset skin allergy did not associate with either disinfection or anxiety, although it did associate with a previous history of allergies. Most medical personnel reported very mild worries and anxieties, which they felt mildly influenced their health. They suspected their skin allergy or airway symptoms correlated with disinfection, whereas our results did not support this. The strong association between COVID-19-related worry to anxiety and newly appearing syndromes suggests the potential influence of worry on both mental and physical conditions amongst medical personnel, requiring further causal investigations and explanations. These findings emphasize the importance of being prepared to support medical personnel through physical and mental health interventions at times of pandemic. It is needed to provide additional support to frontline medical personnel during COVID-19 outbreak to supplement current precautions and care inside and outside of the work environment. Any corresponding early effective responses and interventions aimed at the increased worry may reduce further physical and mental health problems resulting from the COVID-19 outbreaks. This study was limited to a cross-section investigation, which could not identify these subjects nor assess whether worries persisted or lead to chronic mental problems.

## Abbreviations

COVID-19: novel coronavirus disease
SARS-CoV-2: severe acute respiratory syndrome coronavirus 2
C: cluster
CDC: centre for disease control
AHR: airway hyper reactivity
SA: skin allergy
NACIO: sodium hypochlorite disinfectant
CLB: chlorine-based disinfectant
